# The impact of mobility limitations on geriatric rehabilitation outcomes: Positive effects of resistance exercise training (RESORT)

**DOI:** 10.1002/jcsm.13557

**Published:** 2024-09-05

**Authors:** Laure M. G. Verstraeten, Esmee M. Reijnierse, Thom Spoelstra, Carel G. M. Meskers, Andrea B. Maier

**Affiliations:** ^1^ Department of Human Movement Sciences, @AgeAmsterdam, Faculty of Behavioural and Movement Sciences, Amsterdam Movement Sciences Vrije Universiteit Amsterdam The Netherlands; ^2^ Department of Medicine and Aged Care, @AgeMelbourne, The Royal Melbourne Hospital The University of Melbourne Melbourne Victoria Australia; ^3^ Centre of Expertise Urban Vitality, Faculty of Sports and Nutrition Amsterdam University of Applied Sciences Amsterdam The Netherlands; ^4^ Department of Rehabilitation Medicine, Amsterdam Movement Sciences Amsterdam University Medical Center Amsterdam The Netherlands; ^5^ Healthy Longevity Program, Yong Loo Lin School of Medicine National University of Singapore Singapore; ^6^ Centre for Healthy Longevity, @AgeSingapore National University Health System Singapore

**Keywords:** Aged, Dependent ambulation, Physical functional performance, Rehabilitation, Resistance training, Walking

## Abstract

**Background:**

Regaining walking ability is a key target in geriatric rehabilitation. This study evaluated the prevalence of walking ability at (pre‐)admission and related clinical characteristics in a cohort of geriatric rehabilitation inpatients; in inpatients without walking ability, feasibility and effectiveness of progressive resistance exercise training (PRT) were assessed.

**Methods:**

Inpatients within RESORT, an observational, longitudinal cohort of geriatric rehabilitation inpatients, were stratified in those with and without ability to walk independently (defined by Functional Ambulation Classification (FAC) score ≤ 2) at admission; further subdivision was performed by pre‐admission walking ability. Clinical characteristics at admission, length of stay, and changes in physical and functional performance throughout admission were compared depending on (pre‐)admission walking ability. Feasibility (relative number of PRT sessions given and dropout rate) and effectiveness [change in Short Physical Performance Battery, FAC, independence in (instrumental) activities of daily living (ADL/IADL)] of PRT (*n* = 11) in a subset of inpatients without ability to walk independently at admission (able to walk pre‐admission) were investigated compared with usual care (*n* = 11) (LIFT‐UP study).

**Results:**

Out of 710 inpatients (median age 83.5 years; 58.0% female), 52.2% were not able to walk independently at admission, and 7.6% were not able to walk pre‐admission. Inpatients who were not able to walk independently at admission, had a longer length of stay, higher prevalence of cognitive impairment and frailty and malnutrition risk scores, and a lower improvement in independence in (I)ADL compared with inpatients who were able to walk at both admission and pre‐admission. In LIFT‐UP, the relative median number of PRT sessions given compared with the protocol (twice per weekday) was 11 out of 44. There were no dropouts. PRT improved FAC (*P* = 0.028) and ADL (*P* = 0.034) compared with usual care.

**Conclusions:**

High prevalence of inpatients who are not able to walk independently and its negative impact on independence in (I)ADL during geriatric rehabilitation highlights the importance of tailored interventions such as PRT, which resulted in improvement in FAC and ADL.

## Introduction

Walking ability is likely to decline during acute hospitalization of older patients with up to 65% of inpatients losing their ability to walk independently,^1^ defined as a Functional Ambulation Classification (FAC) score ≤ 2 points.^2^ Besides the reason for admission, bed rest and lack of mobilization during hospitalization contribute to the loss of walking ability.^1^ Inability to walk is associated with poor outcome in older adults, such as activities of daily living (ADL) impairment, institutionalization, and death.^3^ Geriatric rehabilitation consists of multidisciplinary interventions, including physical and occupational therapy, to promote independent functioning and muscle strength recovery in older adults after hospitalization.^4^ These interventions, although designed according to the patient's needs, may need further tailoring accounting for walking ability. This includes considering the underlying reason for walking inability such as a lack of strength, endurance, cognition, pain, or a combination of factors.

Up to 50% of geriatric rehabilitation patients are not able to walk independently at admission.^5^, ^6^ But whether walking ability prior to and at admission to geriatric rehabilitation is related to clinical characteristics of inpatients and whether it influences physical and functional performance recovery have not been studied. These insights are important to guide the need for tailored interventions in patients who are unable to walk. Moreover, evidence lacks on the type of interventions needed to regain walking ability, in addition to standard rehabilitation. Several interventions aimed at improving walking ability in community dwelling older adults have been studied, including a combination of resistance, endurance, and balance training,^7^ as well as visual‐related training.^8^ However, feasibility and efficacy of these interventions to improve mobility and independence in ADL in geriatric rehabilitation inpatients without walking ability have not been investigated. Progressive resistance exercise training (PRT) may be beneficial as it has proven to be effective to improve muscle strength and power, balance, gait speed, and independence in ADL in (acutely hospitalized) older adults.^9^, ^10^, ^11^

This study aims to (1) identify the prevalence of independent walking ability at admission to geriatric rehabilitation and pre‐admission in a large cohort; (2) compare admission characteristics, length of stay, and changes in physical and functional performance throughout the admission to geriatric rehabilitation stratified by walking ability at admission and pre‐admission; (3) investigate the feasibility (relative number of PRT sessions given, dropout rate, and related adverse events) and effectiveness of PRT on changes in physical and functional performance from baseline to discharge compared with usual care in a subgroup of inpatients without walking ability at admission.

## Methods

### Study design and population

**Figure 1:**
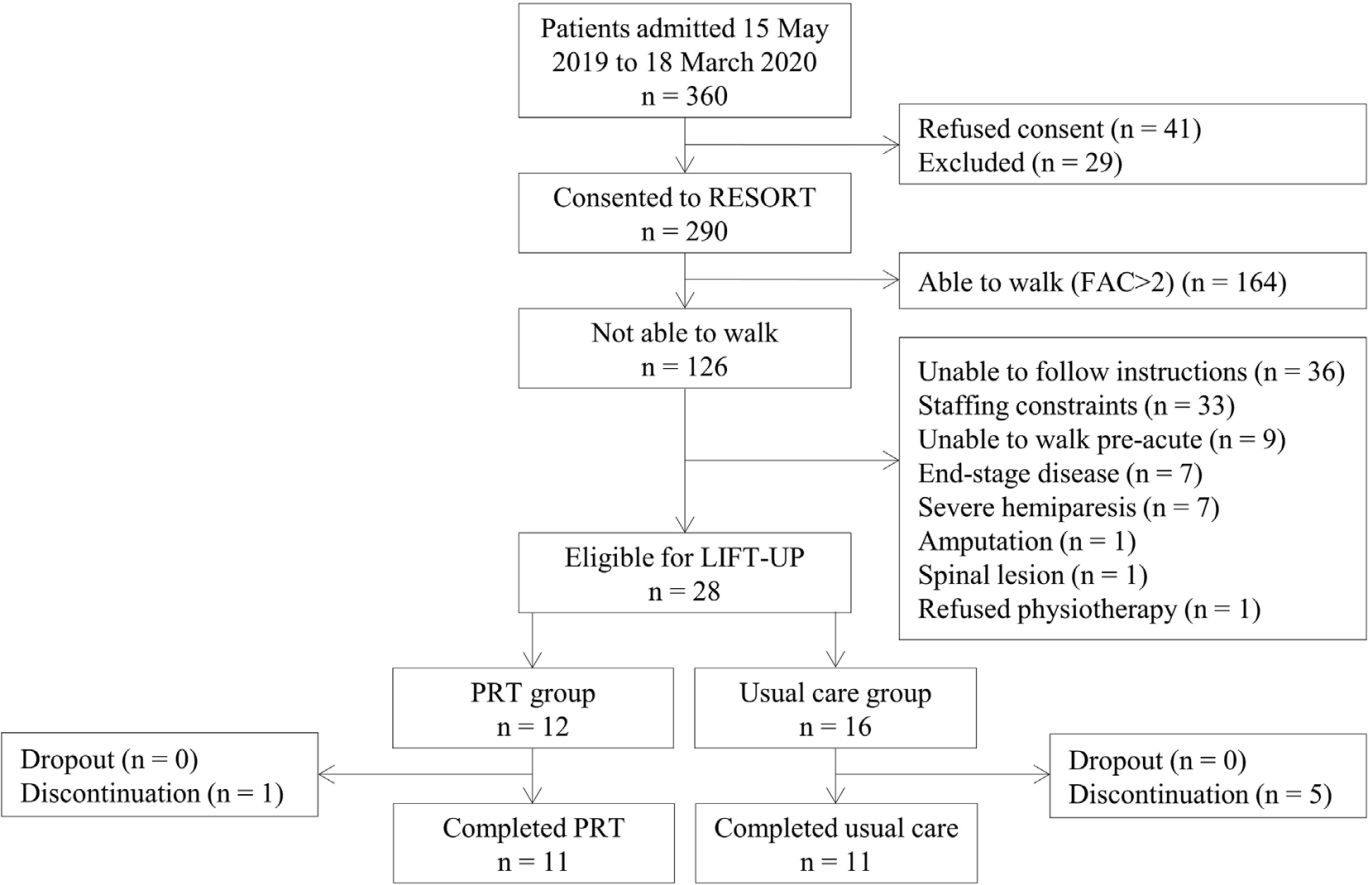
Flowchart of RESORT patients included in LIFT‐UP. FAC, Functional Ambulation Classification; PRT, progressive resistance exercise training.

REStOring health of acutely unwell adulTs (RESORT) is an observational, longitudinal inception cohort of geriatric rehabilitation inpatients admitted to the Royal Melbourne Hospital (Melbourne, Victoria, Australia). Inpatients were assessed using Comprehensive Geriatric Assessment (CGA) performed by physicians, nurses, physiotherapists, occupational therapists, and dietitians within 48 h of admission and 48 h before discharge.^12^ Exclusion criteria were (1) inpatient's inability to provide informed consent and no nominated proxy to consent and (2) palliative admission at admission. The study was approved by the Melbourne Health Human Research Ethics Committee (HREC/17/MH/103) and was conducted in accordance with the Declaration of Helsinki.^13^ Of the 2692 inpatients admitted from 16 October 2017 and discharged by 18 March 2020, 446 were excluded, and 356 refused to consent; a total of 1890 inpatients were included in the RESORT cohort. The FAC was implemented in wave 3, and therefore, for the present analysis, 710 inpatients were included. LIFT‐UP is a feasibility intervention sub study within the RESORT cohort from 15 May 2019 to 18 March 2020, assessing the feasibility and effectiveness of PRT in inpatients without walking ability. LIFT‐UP was conducted on four wards; the PRT programme was enrolled in two of the four wards; the other two wards served as usual care group. Of the 360 admitted inpatients, 164 were able to walk, 98 were excluded for other reasons including inability to follow instructions, end‐stage disease, and severe hemiparesis, and 41 refused to consent to RESORT (Figure 1). Of the 28 inpatients, 12 inpatients were in the PRT group and 16 in the usual care group based on the ward allocation.

### Inpatient characteristics

Age, sex, primary reason for acute admission, and length of stay in geriatric rehabilitation were collected from medical records. Disease burden was assessed by physicians with the 56‐point Cumulative Illness Rating Scale (CIRS)^14^ and 37‐point Charlson co‐morbidity index (CCI).^15^ Cognitive impairment was assessed by physicians and defined by a dementia diagnosis or mild cognitive impairment/minor neurocognitive disorder reported in medical records, CCI or CIRS or by a cognitive score below cuff‐off values for one of the following tests: standardized Mini‐Mental State Examination (sMMSE) < 24 points,^16^ Rowland Universal Dementia Assessment Scale (RUDAS) < 23 points,^17^ or Montreal Cognitive Assessment (MoCA) < 26 points.^18^ Frailty status was assessed by physicians using the Clinical Frailty Scale (CFS) on a scale from 1 (*very fit*) to 9 (*terminally ill*).^19^ Anthropometric measurements were performed by nurses. Standing height was measured barefoot; if the inpatient was unable to stand, knee height was measured with a sliding calliper, and height was estimated using the Chumlea equation.^20^ Body weight was measured using a calibrated weighing scale, weighing chair, or hoist without shoes or heavy clothes. Body mass index (BMI) was calculated by dividing weight by height squared (kg/m^2^). Risk of malnutrition was assessed by nurses using the Malnutrition Screening Tool (MST) on a scale from 0 to 5.^21^ Muscle strength and physical performance were assessed by physiotherapists. Handgrip strength (HGS) was assessed with a handheld dynamometer (JAMAR, Sammons Preston, Inc. Boling‐Brook, IL, USA). Inpatients were asked to squeeze with maximum effort three times on both hands, alternating between right and left, in a seated position with the elbow bent at a 90° angle, adjacent to the body and unsupported.^22^ The maximum score in kilograms was used. The Short Physical Performance Battery (SPPB) includes the standing balance test, the timed chair stand test and the timed 4‐m walk test; score ranges from 0 to 12 points.^23^ Functional performance was assessed by occupational therapists using the Katz index for ADL (0–6 points)^24^ and the Lawton and Brody scale for instrumental ADL (IADL) (0–8 points).^25^

### Walking ability

Walking ability pre‐admission to hospital was assessed as part of a patient survey with the following question: ‘In the month before you were admitted to hospital, were you able to walk (with or without a walking aid)?’. Independent ambulation, here defined as walking ability, at admission to geriatric rehabilitation was assessed by physiotherapists and defined as a FAC score ≤ 2.^2^

### LIFT‐UP

Inclusion criteria were a FAC score ≤ 2 points and being able to follow basic instructions in English or gestures. Exclusion criteria encompassed not being to walk pre‐admission to hospital, weight‐bearing restrictions, inability to follow instructions, end‐stage disease, severe hemiparesis, and spinal lesions. There was no blinding due to the nature of the intervention. Usual care consisted of a maximum of daily physiotherapy sessions, depending on the needs of the inpatient, including functional (walking, transfer from bed/chair, and stair climbing), balance, endurance, and resistance training (variety of upper‐ and lower‐body exercises). The treatment goals were determined together with the patient. There were four types of physiotherapy sessions: case management (multidisciplinary meeting to discuss progress, individual not present), assessment (evaluation), intervention (session alone with physiotherapist), and exercise class (group session). Details on the frequency, duration, and type of physiotherapy received during admission in the RESORT cohort are described elsewhere.^26^ In the PRT group, bi‐daily resistance training sessions were provided in addition to usual care. The PRT programme was a one‐on‐one supervised programme implemented by the treating physiotherapist during the total length of stay in geriatric rehabilitation, designed using the 16‐item checklist Consensus on Exercise Reporting Template (CERT).^27^ The programme consisted of seven exercises (chair raise, hip thrust, glute abduction, lateral pulldown, chest fly, shoulder abduction, and prone hold) performed every weekday split into two 15‐min sessions (morning‐afternoon); one for the lower and one for the upper body. The progression model was designed to be performed on the bed and eventually progressed to complete stance. The progression model incorporated the Oxford Scale, starting with performing the movement assisted and with gravity eliminated with the goal to progress the movement against gravity with resistance. The intensity was assessed by the treating physiotherapist using the rate of perceived exertion (RPE), a scale of 1 (*very easy*) to 10 (*maximal*),^28^ with a desired intensity for each exercise of eight. If eight repetitions of the exercise could be performed at less than RPE eight, the movement was either progressed to the next variation, or resistance was added using elastic bands by Thera‐Band (Hygenic Corp., Akron, USA) with three levels: yellow (low resistance), red (moderate resistance), and green (high resistance).

Feasibility of the PRT programme was assessed as the (1) relative number of PRT sessions given, defined as number of PRT sessions given relative to the total number of sessions planned per protocol (twice per weekday – two assessment sessions); (2) number of dropouts (patients who decided to prematurely withdraw from LIFT‐UP), excluding inpatients who were transferred back to acute hospitalization (considered as discontinuation); and (3) percentage of inpatients with adverse events related to PRET. The outcomes of LIFT‐UP were the changes in SPPB, FAC, ADL, and IADL scores from baseline (inclusion LIFT‐UP) at discharge.

### Statistical analysis

Median and interquartile ranges (IQR) were reported for non‐normally distributed continuous variables and frequencies (*n*) with percentages (%) for categorical variables. Changes in SPPB, FAC, ADL, and IADL during geriatric rehabilitation were calculated by subtracting the admission from the discharge score. Differences in characteristics dependent on walking ability were assessed with Kruskal–Wallis tests for continuous/ordinal variables and chi‐square tests for categorical variables. Bonferroni corrections were applied for pairwise comparisons resulting in *P* values < 0.008 to be considered statistically significant (six comparisons). For LIFT‐UP, inpatients who discontinued the study (transfer to acute hospitalization) were excluded from the analyses. Differences in baseline characteristics between the PRT and the usual care group were assessed with Mann–Whitney *U* tests for continuous/ordinal variables and chi‐square tests for categorical variables. Within‐group differences in SPPB, FAC, ADL, and IADL scores between baseline and discharge were assessed with Wilcoxon‐signed rank tests and between‐group differences with Mann–Whitney *U* tests. *P*‐values of <0.05 were considered statistically significant. All statistical analyses were performed using the Statistical Package for the Social Sciences (IBM SPSS Advanced Statistics 27.0).

## Results

Median length of stay (*n* = 710) was 20 days [IQR: 13–32], and median SPPB, ADL, and IADL scores at admission were 1 [IQR: 0–4], 2 [IQR: 1–2], and 1 [IQR: 0–2], respectively (Table 1). In the LIFT‐UP study (*n* = 28), one inpatient discontinued (transfer to acute hospitalization) in the PRT group and five in the usual care group. The PRT group (*n* = 11) had a lower BMI, and a lower prevalence of cognitive impairment compared with the usual care group (*n* = 11) (Table 1).

**Table 1:** Characteristics at admission to geriatric rehabilitation of inpatients included in RESORT (*n* = 710) and LIFT‐UP (*n* = 22)

	RESORT (*n* = 710)		LIFT‐UP (*n* = 22)				
			PRT group (*n* = 11)		Usual care group (*n* = 11)		
Characteristics	*n*	Total	*n*	Total	*n*	Total	*P* ^a^
Age (years)	710	83.5 [77.8–89.4]	11	85.9 [79.5–92.2]	11	79.5 [77.4–86.2]	0.193
Female, *n* (%)	710	412 (58.0)	11	9 (81.8)	11	6 (54.5)	0.170
Primary reason for acute admission, *n* (%)	710		11		11		0.363
Musculoskeletal		338 (47.6)		7 (63.6)		5 (45.5)	
Neurological		100 (14.1)		1 (9.1)		2 (18.2)	
Respiratory		53 (7.5)		1 (9.1)		0 (0.0)	
Cardiac		50 (7.0)		0 (0.0)		1 (9.1)	
Other		169 (23.8)		2 (18.2)		2 (18.2)	
Length of stay^c^ (days)	710	20 [13–32]	11	22 [17–32]	11	38 [17–49]	0.151
CIRS score [0–56] (points)	710	12 [8–16]	11	15 [10–19]	11	15 [11–17]	1.000
CCI score [0–37] (points)	710	2 [1–3]	11	2 [2–4]	11	3 [1–4]	0.847
Cognitive impairment, *n* (%)	710	466 (65.6)	11	5 (45.5)	11	11 (100)	**0.011**
Clinical Frailty Scale [1–9] (points)	652	6 [5–7]	10	6 [6–7]	11	6 [6–7]	0.426
Body mass index (kg/m^2^)	694	25.7 [22.2–29.8]	11	23.4 [20.4–27.2]	11	30.6 [27.0–35.0]	**0.013**
Malnutrition Screening Tool [0–5] (points)	695	1 [0–2]	11	2 [1–4]	11	0 [0–2]	0.056
Handgrip strength (kg)	547	16.0 [11.5–22.0]	8	15.5 [8.0–19.5]	10	16.0 [11.0–19.0]	0.829
Female	291	13.0 [9.0–17.6]	7	14.0 [8.0–18.0]	5	12.0 [8.5–17.5]	^b^
Male	241	21.7 [16.0–26.0]	1	28.0 [28.0–28.0]	5	16.0 [14.5–24.0]	^b^
**Physical performance**							
SPPB score [0–12] (points)	655	1 [0–4]	9	0 [0–0]	11	0 [0–1]	0.882
FAC score [0–5] (points)	710	2 [0–3]	11	1 [0–2]	11	1 [0–2]	0.847
**Functional performance**							
Katz‐ADL score [0–6] (points)	709	2 [1–2]	11	1 [1–1]	11	1 [1–2]	0.562
Lawton‐IADL score [0–12] (points)	709	1 [0–2]	11	1 [0–2]	11	1 [0–1]	0.300

All results are presented as median [IQR] unless otherwise indicated. Between‐group difference significant at the 5% level, indicated in bold.

(I)ADL, (instrumental) activities of daily living; CCI, Charlson co‐morbidity index; CIRS, Cumulative Illness Rating Scale; FAC, Functional Ambulation Classification; IQR, interquartile range; PRT, progressive resistance exercise training; SPPB, Short Physical Performance Battery.

^a^ The difference between the PRT group and control group of the LIFT‐UP study.

^b^ No *P*‐value presented because of the small sample size.

^c^ In geriatric rehabilitation.

### Walking ability

**Figure 2:**
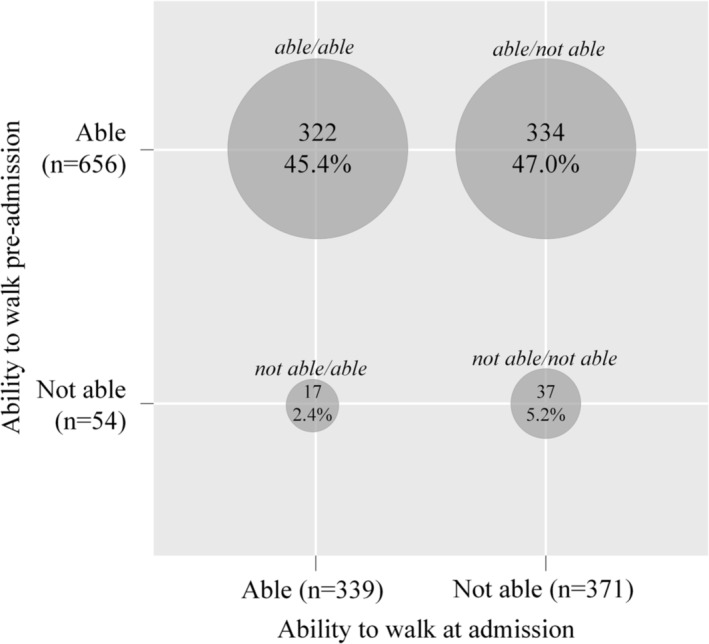
Prevalence of walking ability pre‐admission (before acute hospital admission) and at admission to geriatric rehabilitation (*n* = 710).

Overall, 7.6% (*n* = 54) of inpatients were not able to walk independently pre‐admission, and 52.2% (*n* = 371) were not able to walk independently at admission. When combining pre‐admission and admission, 45.4% (*n* = 322) of inpatients were able to walk at both pre‐admission and admission (able/able), 2.4% (*n* = 17) were not able to walk independently pre‐admission but were able at admission (not able/able), 47.0% (*n* = 334) were able to walk independently pre‐admission but not at admission (able/not able), and 5.2% (*n* = 37) were not able to walk independently at both pre‐admission and admission (not able/not able) (Figure 2).

### Clinical characteristics, length of stay, and changes in physical and functional performance stratified by walking ability

Inpatients who were not able to walk independently at admission (able/not able – not able/not able groups) had a longer length of stay, worse cognition, higher frailty and malnutrition risk, lower independence in IADL at admission, and a lower improvement in independence in IADL during admission compared with inpatients who were able to walk at admission and pre‐admission (able/able group) (Table 2). Inpatients who were not able to walk independently at admission but able to walk pre‐admission (able/not able group) had a higher improvement in mobility (FAC) during admission compared with inpatients who were able to walk at both admission and pre‐admission (able/able group). Inpatients who were not able to walk independently at both admission and pre‐admission (not able/not able group) had a lower improvement in independence in ADL during admission compared with inpatients who were able to walk at both admission and pre‐admission (able/able group). There were no differences in age, sex, reason for admission, morbidity, BMI, handgrip strength, and change in physical performance between groups.

**Table 2:** Clinical characteristics at admission to geriatric rehabilitation and change in physical and functional performance during admission in the RESORT cohort, stratified by walking ability (*n* = 710)

	Walking ability								
	Able/able^a^ (*n* = 322)		Not able/able^a^ (*n* = 17)		Able/not able^a^ (*n* = 334)		Not able/not able^a^ (*n* = 37)		
Characteristics	*n*	Total	*n*	Total	*n*	Total	*n*	Total	*P* ^b^
Age (years)	322	83.1 [77.4–88.9]	17	80.2 [76.9–84.9]	334	84.1 [78.8–90.0]	37	85.3 [79.2–88.6]	0.076
Female, *n* (%)	322	178 (55.3)	17	15 (88.2)	334	197 (59.0)	37	22 (59.5)	0.057
Primary reason for acute admission, *n* (%)	322		17		334		37		0.083
Musculoskeletal		145 (45.0)		9 (52.9)		169 (50.6)		15 (40.5)	
Neurological		34 (10.6)		2 (11.8)		54 (16.2)		10 (27.0)	
Respiratory		30 (9.3)		0 (0.0)		20 (6.0)		3 (8.1)	
Cardiac		33 (10.2)		1 (5.9)		15 (4.5)		1 (2.7)	
Other		80 (24.8)		5 (29.4)		76 (22.8)		8 (21.6)	
Length of stay^c^ (days)	322	15 [10–23]^d^ ^,^ ^e^	17	19 [10–24]	334	24 [17–39]	37	26 [14–41]	**<0.001**
CIRS [0–56] (points)	322	11 [7–15]	17	14 [8–16]	334	12 [8–16]	37	11 [8–14]	0.117
CCI [0–37] (points)	322	2 [1–3]	17	2 [1–4]	334	2 [1–4]	37	2 [1–4]	0.112
Cognitive impairment, *n* (%)	322	196 (60.9)^d^ ^,^ ^e^	17	9 (52.9)	334	233 (69.8)	28	21 (75.7)	**0.035**
Clinical Frailty Scale [1–9] (points)	289	6 [4–6]^d^ ^,^ ^e^	15	5 [4–6]	314	7 [6–7]	34	6 [5–7]	**<0.001**
Body mass index (kg/m^2^)	317	26.3 [22.7–30.1]	17	27.2 [23.0–31.3]	324	25.3 [21.7–29.0]	36	25.1 [22.9–31.9]	0.106
Malnutrition Screening Tool [0–5] (points)	317	0 [0–2]^d^ ^,^ ^e^	17	1 [0–2]	325	1 [0–2]	36	2 [0–2]	**<0.001**
Handgrip strength (kg)	269	18.0 [12.0–22.5]	15	12.0 [8.0–18.0]	241	15.0 [10.0–20.5]	22	14.0 [6.0–22.0]	0.008
**Physical performance**									
SPPB score at admission [0–12] (points)	291	4 [2–6]^d^ ^,^ ^e^	16	2 [1–4]^d^ ^,^ ^e^	313	0 [0–0]	35	0 [0–0]	**<0.001**
Change from admission to discharge	251	1 [0–3]	12	1 [0–2]	250	1 [0–3]	31	0 [0–3]	0.345
FAC score [0–5] (points)									
Change from admission to discharge	302	1 [0–1]^d^	14	1 [0–1]	298	2 [0–3]	33	1 [0–3]	**<0.001**
**Functional performance**									
Katz‐ADL score [0–6] (points)	322	2 [1–4]^d^ ^,^ ^e^	17	2 [2–3]^d^ ^,^ ^e^	333	1 [0–2]	37	1 [0–2]	**<0.001**
Change from admission to discharge	314	2 [1–3]^e^	16	2 [1–3]	305	1 [0–3]	36	0 [0–2]	**0.005**
Lawton‐IADL score [0–12] (points)	322	1 [1–2]^d^ ^,^ ^e^	17	1 [0–3]	333	1 [0–2]	37	0 [0–1]	**<0.001**
Change from admission to discharge	315	2 [0–4]^d^ ^,^ ^e^	16	2 [0–3]	305	1 [0–2]	36	0 [0–2]	**<0.001**

All results are presented as median [IQR] unless otherwise indicated. Between‐group difference significant at the 0.8% level, indicated in bold.

^a^ Able or not able before the backlash refers to walking ability pre‐acute admission and after the backlash to walking ability at admission.

^b^ Statistical difference between the four groups; pairwise comparisons adjusted with Bonferroni correction (*P* < 0.008).

^c^ In geriatric rehabilitation.

^d^ Significant difference from the ‘able/not able’ group.

^e^ Significant difference from the ‘not able/not able’ group.

### Feasibility and effectiveness of progressive resistance exercise training in the LIFT‐UP study

The duration of the intervention varied between 8 and 48 days (until discharge) and relative median number of PRT sessions given compared with protocol was 11 out of 44 (Table 3). There were no dropouts in both the PRT and usual care group. In the PRT group (*n* = 11), SPPB, FAC, ADL, and IADL scores significantly improved between baseline and discharge (Table 4). In the usual care group (*n* = 11), SPPB and FAC significantly improved between baseline and discharge, and ADL and IADL scores did not improve. PRT improved change in FAC (*P* = 0.028) and ADL (*P* = 0.034) between baseline and discharge compared with usual care, but not SPPB (*P* = 0.837) and IADL (*P* = 0.243).

**Table 3:** Relative number of progressive resistance exercise training sessions given compared with protocol (two sessions per day) for the LIFT‐UP study (*n* = 11)

Patient^a^	Study duration (days)	Weekend Days	Number of PRT sessions given			Relative number of sessions
			Morning	Afternoon	Total	Given/maximum per protocol^b^
1	13	2	8	3	11	11/20
2	8	2	3	2	5	5/10
3	16	4	4	6	10	10/22
4	32	10	13	6	19	19/42
5	48	14	19	4	23	23/66
6	31	8	6	5	11	11/44 (median)
7	9	2	3	0	3	3/12
8	14	4	3	0	3	3/18
9	27	8	5	0	5	5/36
10	18	4	1	0	1	1/26
11	24	6	1	0	1	1/34

PRT, progressive resistance exercise training.

^a^ Ordered in descending order of relative number of PRT sessions given.

^b^ Maximum number of sessions per protocol = [(study duration (days) − number of weekend days)*2] − 2.

**Table 4:** Effect of progressive resistance exercise training on physical and functional performance change during admission compared with usual care in geriatric rehabilitation inpatients for the LIFT‐UP study (*n* = 22)

Outcome	PRT group (*n* = 11)				Usual care group (*n* = 11)				Difference
	Baseline	Discharge	Change^a^	*P* ^b^	Baseline	Discharge	Change^a^	*P* ^b^	*P* ^c^
**Physical performance**									
SPBB score [0–12] (points)	0 [0–1]	3 [1–6]	3 [0–5]	**0.041**	0 [0–1]	3 [0–6]	3 [0–6]	**0.027**	0.837
FAC score [0–5] (points)	1 [0–2]	4 [3–4]	2 [2–4]	**0.003**	1 [0–2]	2 [0–4]	0 [0–2]	**0.042**	**0.028**
**Functional performance**									
Katz‐ADL score [0–6] (points)	1 [1–1]	5 [1–5]	3 [1–4]	**0.007**	1 [1–2]	1 [1–4]	0 [0–2]	0.168	**0.034**
Lawton‐IADL score [0–12] (points)	1 [0–2]	2 [1–5]	1 [1–4]	**0.011**	1 [0–1]	1 [0–4]	0 [0–3]	0.078	0.243

All results are presented as median [IQR]. Significant at the 5% level, indicated in bold.

(I)ADL, (instrumental) activities of daily living; FAC, Functional Ambulation Classification; IQR, interquartile range; PRT, progressive resistance exercise training; SPPB, Short Physical Performance Battery.

^a^ Change in score from baseline to discharge.

^b^ Within‐group difference between baseline and discharge scores.

^c^ Between‐group (PRT vs. usual care) difference in change in score from baseline to discharge.

## Discussion

In this cohort of geriatric rehabilitation inpatients, half were not able to walk independently at admission. (Pre‐)admission walking ability had a significant impact on clinical characteristics at admission and improvements observed during rehabilitation. Notably, inpatients who were not able to walk independently at admission had a longer length of stay, worse cognition, higher frailty and malnutrition risk, and a lower improvement in independence in (I)ADL compared with inpatients who were able to walk at (pre‐)admission. Progressive resistance training improved changes in FAC and independence in ADL but not in SPPB and independence in IADL between baseline and discharge when compared with usual care in inpatients without walking ability.

Previous studies in older adults at discharge from acute hospitalization showed lower prevalence of inability to walk (16.8%)^29^ and similar prevalence (45.0%)^30^ compared with this cohort. This may be due to difference in assessment as the former study used subjective patient reports, which may overestimate walking ability.^31^ Moreover, lower walking ability can be expected in geriatric rehabilitation compared with acute hospitalization as patients who are able to walk are more likely to be discharged home directly after acute admission. Previous studies in geriatric rehabilitation also showed around 50% of patients were not able to walk at admission.^5^, ^6^ The decline in walking ability between pre‐admission and admission is in line previous studies^1^ although inability to walk pre‐admission is commonly solely described by the use of a walking aid.^32^ Future research should investigate whether there are specific risk factors of prolonged inability to walk.

Similarly to our findings, low physical function (Barthel Index/Katz‐ADL/gait speed/balance) at admission^33^ and reduced pre‐fracture mobility (use of a walking aid)^34^ were associated with a longer length of stay in hospitalized older adults. Moreover, poor mobility and pre‐admission loss of independence in ADL have shown to be predictors of functional decline during acute hospitalization in older adults.^35^, ^36^ This highlights the importance of tailored interventions in patients without walking ability to improve function recovery during rehabilitation, as this is crucial to facilitate discharge home. The higher improvement in mobility (FAC) in inpatients who are not able to walk is likely to be caused by a ceiling effect as inpatients who can already walk at admission will have less room for improvement. Specifically, physical therapy interventions in geriatric rehabilitation are designed to enable patients to achieve a satisfactory level of independent functioning following discharge; walking outside and climbing stairs may not be trained if not deemed necessary. In line with our findings, hip fracture patients with worse pre‐fracture nutritional status assessed by the Mini Nutritional Assessment were less likely to regain their pre‐fracture mobility.^37^ Improving nutritional interventions prior and during rehabilitation may thus play a key role to promote walking ability. Similarly to our results, in hospitalized older adults, cognitive impairment had a negative impact on mobility recovery (Tinetti Performance‐Oriented Mobility Assessment)^38^ and lead to an increased risk of functional decline.^39^ Cognitive impairment therefore also needs to be considered when tailoring rehabilitation interventions to recover walking ability. Previous studies did not take pre‐admission walking ability into account. The present results show most differences in clinical characteristics occur based on admission walking ability. However, the small group size of inpatients who were not able to walk pre‐admission may have influenced the results.

Interestingly, while inability to walk independently at admission impeded improvement in ADL, PRT in patients who were unable to walk at admission showed to significantly improve change in ADL and FAC during geriatric rehabilitation admission compared with usual care. Although further research is needed with a larger sample size, this shows that the addition of PRT to the rehabilitation plan of patients who are unable to walk may improve their recovery. Systematic reviews investigating the effect of PRT in older adults after a hip fracture surgery,^7^ community‐dwelling older adults,^8^ and nursing homes residents^11^ have also shown significant improvements in muscle strength, functional performance, and physical function. PRT twice daily was not feasible in most inpatients. Lack of patients' knowledge on the importance of PRT for muscle strength,^40^ other interventions and medical appointments, and visitors may have limited feasibility. Understanding the reasons why PRT sessions were not given (patient and not patient related) is needed to assess feasibility further.

### Strengths and limitations

To the best of our knowledge, this is one of the first studies assessing (pre‐) admission walking ability and thereto related clinical characteristics in a large cohort of geriatric rehabilitation inpatients. Furthermore, all inpatients were assessed using a CGA, which promotes validity and standardization of measurements in older patients. A limitation of this study is the self‐reported assessment of walking ability pre‐admission to hospital, which may have overestimated walking ability.^31^ Furthermore, the LIFT‐UP study consisted of a small sample size due to the nature of an exploratory study and BMI and cognitive impairment prevalence were significantly higher in the PRT group compared with the usual care group, warranting caution with results interpretation. Finally, this was a single‐site study, which could limit generalisability to other geriatric rehabilitation settings and countries. Further research is therefore needed to inform clinical practice.

## Conclusions

The prevalence of inability to walk independently at admission was high and associated with a longer length of stay, worse cognition, and nutritional status at admission, as well as worse functional performance improvement during admission compared with inpatients who were able to walk, which emphasizes the importance of interventions tailored for walking ability. Progressive resistance exercise training improved FAC and independence in ADL but not SPPB and IADL independence in the feasibility intervention study. Further research with a larger sample size is needed.

## Funding

This work was supported by an unrestricted grant of the University of Melbourne received by Prof. Andrea B. Maier and the Medical Research Future Fund (MRFF) provided by the Melbourne Academic Centre for Health (MACH). This work is also part of a collaboration project co‐funded by the PPP Allowance made available by Health~Holland (grant number TKI‐LSHM19069‐H049), Top Sector Life Sciences & Health, to stimulate public‐private partnerships, and Top Sector Agri & Food (grant number LWV19287). The collaboration project also includes an in‐cash and in‐kind contribution from Danone Nutricia Research.

## Conflict of interest

Andrea B. Maier reports grants from Danone Nutricia Research outside the conduct of the study. The other authors declare that they have no conflicts of interest.
